# Gene Expression Profiles of Metabolic Aggressiveness and Tumor Recurrence in Benign Meningioma

**DOI:** 10.1371/journal.pone.0067291

**Published:** 2013-06-28

**Authors:** Eva Serna, José Manuel Morales, Manuel Mata, José Gonzalez-Darder, Teresa San Miguel, Rosario Gil-Benso, Concha Lopez-Gines, Miguel Cerda-Nicolas, Daniel Monleon

**Affiliations:** 1 Unidad Central de Investigación en Medicina, Universitat de Valéncia, Valencia, Spain; 2 Servicio de Neurocirugía, Hospital Clínico Universitario de Valencia, Valencia, Spain; 3 Departamento de Patología, Universitat de Valencia, Valencia, Spain; 4 Centro de Investigación Biomédica en Red de Enfermedades Respiratorias (CIBER-RES), Madrid, Spain; 5 Fundación de Investigación del Hospital Clínico Universitario de Valencia/Instituto de Investigacion Sanitaria Clinico Valencia (INCLIVA), Valencia, Spain; Centro di Riferimento Oncologico, IRCCS National Cancer Institute, Italy

## Abstract

Around 20% of meningiomas histologically benign may be clinically aggressive and recur. This strongly affects management of meningioma patients. There is a need to evaluate the potential aggressiveness of an individual meningioma. Additional criteria for better classification of meningiomas will improve clinical decisions as well as patient follow up strategy after surgery. The aim of this study was to determine the relationship between gene expression profiles and new metabolic subgroups of benign meningioma with potential clinical relevance. Forty benign and fourteen atypical meningioma tissue samples were included in the study. We obtained metabolic profiles by NMR and recurrence after surgery information for all of them. We measured gene expression by oligonucleotide microarray measurements on 19 of them. To our knowledge, this is the first time that distinct gene expression profiles are reported for benign meningioma molecular subgroups with clinical correlation. Our results show that metabolic aggressiveness in otherwise histological benign meningioma proceeds mostly through alterations in the expression of genes involved in the regulation of transcription, mainly the LMO3 gene. Genes involved in tumor metabolism, like IGF1R, are also differentially expressed in those meningioma subgroups with higher rates of membrane turnover, higher energy demand and increased resistance to apoptosis. These new subgroups of benign meningiomas exhibit different rates of recurrence. This work shows that benign meningioma with metabolic aggressiveness constitute a subgroup of potentially recurrent tumors in which alterations in genes regulating critical features of aggressiveness, like increased angiogenesis or cell invasion, are still no predominant. The determination of these gene expression biosignatures may allow the early detection of clinically aggressive tumors.

## Introduction

Meningiomas are common Central Nervous System tumors that arise from the leptomeningeal covering of the brain and spinal cord and account for around 20% of all central nervous system tumors. These tumors show a remarkably wide biologic and histological heterogeneity. They are often considered benign tumors curable by surgery. The current standards for diagnosis of meningiomas are clinical and pathological findings. The World Health Organization (WHO) classifies meningiomas into three histological grades: grade I (benign), grade II (atypical), and grade III (anaplasic) in accordance with the clinical prognosis [1]. Atypical and anaplasic meningiomas constitute the most clinically aggressive forms and often recur. However, around 20% of meningiomas histologically benign may also be clinically aggressive and recur [2]. In absolute numbers, most recurrent meningiomas correspond to histological benign tumors. This strongly affects management and follow up strategy of meningioma patients. There is a need to evaluate the potential aggressiveness of an individual meningioma.

Genetic characterization of meningiomas has some value in the sub classification and management of meningiomas. The genesis of meningiomas has been associated with loss of genetic material on chromosome 22. Monosomy of this chromosome is the most common genetic alteration in meningioma and was one of the first cytogenetic alterations described in solid tumors [3], [4]. Loss of 1p and alterations in chromosome 14 are present in many atypical meningioma [5]. Losses in 6q, 10 and 18q and gains on 1q, 9q, 12q, 15q, 17q and 20q are also common in atypical meningioma [6]. Recent studies show that benign meningiomas with alterations in chromosome 14, among others, may be clinically aggressive and recur [7]. Although these genetic markers provide the basis for meningioma subclassification, the determination of phenotypic markers of aggressiveness before clinical progression is essential for choosing the follow up strategy of the individual meningioma patient. The next generation of hallmarks of cancer include reprogramming of energy metabolism as a major driver of tumor progression [8]. This reprogramming affects metabolic pathways essential for tumor growth and survival, like the Kennedy pathway, the anaerobic glycolysis, the fatty acids oxidation and the production of cell antioxidants. A recent study shows that the measurement of a metabolic phenotype in primary tumor tissue specimens simultaneously to the histopathology analysis may allow the early detection of metabolically aggressive tumors. Metabolically aggressive tumors exhibit higher levels of metabolites associated to the aforementioned pathways, among others. Based on this metabolic aggressiveness, it is possible to define new molecular subgroups of benign meningioma [9].

Gene expression profiling by oligonucleotide microarrays allow the screening of thousands of genes simultaneously. Several studies reported microarray analysis of meningiomas in the past with different aims, microarray platforms and statistical approaches [10]–[13]. The result of such a variety of approaches is no shared genes deregulated in all these studies. One possible reason for these results may be the existence of different molecular subgroups of meningioma. The intrinsic variation of aggressiveness and genetic alterations within benign meningioma highly impacts the statistical results of molecular studies on meningioma. Perez-Magan et al. compared previously reported gene expression profiles associated with meningioma progression and detected a profile potentially associated with recurrence. The final candidate genes were extracted from 4 original tumors that recurred later out of a total of 44 meningiomas with microarray gene expression data. In this context, the use of molecular phenotypic criteria for the determination of meningioma subgroups would help in obtaining more robust gene expression profiles. The determination of these profiles combined with other markers of molecular aggressiveness may also aid in determining the aggressiveness of surgical resection and the necessity of combined radiation therapy.

The aim of this study was to determine the relationship between gene expression profiles and new metabolic subgroups of benign meningioma. We obtained molecular profiles in 40 benign and 14 atypical meningioma tissue samples by performing metabolic, cytogenetic and gene expression analysis. Based on recently published criteria for molecular classification, we detected benign meningioma molecular subgroups and explored differential gene expression between them.

## Methods

### Tumor Material

Fifty-four human meningiomas biopsies were obtained at the Department of Neurosurgery of the Clinical University Hospital of Valencia. This study was reviewed and approved by the Clinical University Hospital of Valencia.ethics committee. Patients gave written informed consent for participating in the study. During surgery, most of the resected tissue was sent for routine histological analysis, some fresh tissue was kept in culture media for cytogenetic studies, and the remainder was immediately put in cryogenic vials and snap-frozen in liquid nitrogen. All snap-frozen samples were stored in a freezer at −80 C until further analysis. All samples used for histopathological examination were fixed in neutral-buffered formalin, embedded in paraffin, sectioned and stained with hematoxylin-eosin. Tumors were classified according to the 2007 WHO Histological Classification [1]. Grade II meningioma were classified according to previously published criteria [2], including high mitotic index and three out of five markers of atypia.

### Cytogenetics

Cytogenetic analyses were performed by short-term culture of the tumors. Fresh tumor samples were disaggregated with 2 mg/mL of collagenase II. The cells were seeded in flasks using RPMI-1640 medium supplemented with 20% fetal bovine serum, L-glutamine, and antibiotics. The cells were processed after 72 h of culture by a standard technique [5]. Air dried slides were banded by trypsin-Giemsa. Karyotypic analyses were performed according to International System for Human Cytogenetics (ISCN) [14].

### Fluorescence In Situ Hybridization

The samples of meningioma used for Florescence In-itu Hybridization (FISH) analysis were studied by tissue microarrays (TMA). We removed four 0.6-mm cores from the corresponding areas on the paraffin block in each case, using the Beecher Instruments Manual Tissue ArrayerI (Beecher Instruments, Sun Prairie, WI, USA). For the investigation of chromosome abnormalities by iFISH the probes LSI 22q12, LSI 1p36/LSI 1q25 and LSI t(11;14) IGH/CCND1 (Vysis Inc; Downers Grove,IL) were used.

Hybridizations were performed according to the instructions that accompany the probe. Counterstaining of nuclei was carried out using 4′,6-diamidino-2-phenylindole (DAPI). The fluorescent signal was detected using a photomicroscope Axioplan 2 and Axiophot 2 (Zeiss) equipped with a set of the appropriate filters. For each hybridization, green and orange signals were counted in the four regions of a total of 100–200 non-overlapping nuclei. An interpretation of deletion or imbalance was made when >20% of the nuclei harbored these alterations [15], [16]. Cutoffs for deletions were based on the frequencies of signals for the same probes in non-neoplastic brain controls (median+-3 ) and ranged from 14 to 21% for chromosome 1, from 16 to 22% for chromosome 14, and from 15 to 20% for monosomy 22. We considered deletion when appeared one or less signal for chromosome with respect to the signal of control (ratio 0/1, 0/2, 1/2, 1/3…) and we considered normal with the probes used present a ratio 2/2.

### Metabolic Profiles

Metabolic profiles were measured in 54 meningioma samples using Nuclear Magnetic Resonance (NMR) spectroscopy as described elsewhere [9]. In summary, the tissue sample for NMR spectroscopy was split from the whole frozen tumoral mass submerged in liquid nitrogen. The mean sample weight was 26±10 mg. All NMR spectra were recorded in a Bruker Avance DRX 600 spectrometer (Valencia, Spain) operating at a ^1^H frequency of 600.13 MHz. For all experiments, samples were spun at 5000 Hz to keep the rotation sidebands out of the acquisition window. In order to minimize the effects of tissue degradation, which would alter the metabolite composition of the biopsy, all NMR spectra were acquired at this temperature of 277K. A single-pulse pre-saturation experiment was acquired in all the samples. Immediately after the measurement, the samples were fixed in formalin for subsequent histopathological examination and for tumor content assessment by an expert pathologist. All NMR spectra were processed using MNova 5.3 (MestreLab S.A., Santiago de Compostela, Spain) and transferred to MATLAB (MathWorks Inc, 2006) using in-house scripts for data analysis. Spectral signal integration by peak-fitting algorithms over relevant resonances provided relative levels of the corresponding metabolites. Only those signals with peak-fitting residual error lower than 10% were used in the study. Statistical significance of the differences was calculated by the Sudent’s t-test. The level of significance was set at p≤0.05.

### RNA Extraction and RNA Integrity Control

Total RNA from frozen tissue of different groups was extracted using a mirVANA miRNA Isolation Kit (Ambion, AMBION INC•THE RNA COMPANY, Austin) and concentration was quantified with the Genequant Pro Classic spectrophotometer by measuring the extinction at 260 nm. Additionally, the OD260/230 and the OD260/280 ratio showing RNA purity were examined. The quality was verified by using the Agilent 2100 BioAnalyzer with ‘‘Eukaryote total RNA Nano Assay” (Agilent Technologies).The RNA integrity number (RIN) served as RNA integrity parameter (selection criteria RIN ≥7.0).

### Gene Expression Microarray Analysis

The GeneChip Human Genome U133 plus2.0 Array containing over 47,000 transcripts and variants, and represent over 39,000 well-characterized human genes (Affymetrix, Santa Clara, CA, USA) was used for microarray analysis. The fragmentation of biotinylated cRNA derived from 300 ng of total RNA was used to hybridize to GeneChips. The hybridization cocktail was incubated overnight at 45°C while rotating in a hybridization oven. After 16 h of hybridization, the cocktail was removed and the arrays were washed and stained in an Affymetrix GeneChip Fluidics Station 450, according to the Affymetrix-recommended protocol. The distribution of fluorescent material on the array was obtained using GeneChip Scanner 3000 7G (Affymetrix, Santa Clara, CA, USA). GeneChip Operating Software supplied by Affymetrix was used to generate.CEL files and were analyzed and statistically filtered using software Partek Genomic Suite 6.4 (Partek Inc., St. Louis, MO, USA). Input files were normalized with the robust multi-chip average (RMA) algorithm for gene array. To narrow the list of relevant genes, we applied a restrictive filtering algorithm using a combined criterion, which required both a fold change absolute value of 2 or higher and a statistical significance of p<0.005 between subgroups. A Benjamini–Hochberg step-up false discovery rate procedure was also used with a final maximum FDR value of 0.005 (See Table 1).

**Table 1:** Fold-change and statistical significance for genes differentially expressed between benignA and benignB meningioma subgroups.

Probeset ID	Gene Title	Gene Symbol	Chromosomal Location	p-value	FDR	FC
						
228904_at	homeobox B3	HOXB3	chr17q21.3	0,0018	0,0034	9,89
204424_s_at	LIM domain only 3 (rhombotin-like 2)	LMO3	chr12p12.3	0,0040	0,0044	8,35
205366_s_at	homeobox B6	HOXB6	chr17q21.3	0,0030	0,0042	6,23
235521_at	homeobox A3	HOXA3	chr7p15-p14	0,0029	0,0042	5,34
213931_at	inhibitor of DNA binding 2, dominant negative helix-loop-helix protein///inhib	ID2///ID2B	chr2p25///chr3p14.2	0,0008	0,0025	3,16
214457_at	homeobox A2	HOXA2	chr7p15-p14	0,0027	0,0042	2,45
239791_at	similar to hCG2042068	LOC100130740	chr17q21.32	0,0010	0,0025	2,31
1557051_s_at	–	–	–	0,0031	0,0042	2,22
208470_s_at	haptoglobin///haptoglobin-related protein	HP///HPR	chr16q22.1	0,0044	0,0046	2,22
232054_at	protocadherin 20	PCDH20	chr13q21	0,0034	0,0042	2,12
239966_at	–	–	–	0,0010	0,0025	2,07
217511_at	Kazal-type serine peptidase inhibitor domain 1	KAZALD1	chr10q24.31	0,0027	0,0042	2,05
242379_at	–	–	–	0,0041	0,0044	2,03
238768_at	chromosome 2 open reading frame 68	C2orf68	chr2p11.2	0,0017	0,0034	−2,01
205964_at	zinc finger protein 426	ZNF426	chr19p13.2	0,0003	0,0018	−2,01
205037_at	RAB, member of RAS oncogene family-like 4	RABL4	chr22q13.1	0,0009	0,0025	−2,03
235729_at	zinc finger protein 514	ZNF514	chr2q11.1	0,0004	0,0020	−2,03
204963_at	sarcospan (Kras oncogene-associated gene)	SSPN	chr12p11.2	0,0031	0,0042	−2,06
211651_s_at	laminin, beta 1	LAMB1	chr7q22	0,0011	0,0025	−2,08
225330_at	insulin-like growth factor 1 receptor	IGF1R	chr15q26.3	0,0011	0,0025	−2,09
226231_at	–	–	–	0,0010	0,0025	−2,09
233576_at	3-hydroxymethyl-3-methylglutaryl-Coenzyme A lyase-like 1	HMGCLL1	chr6p12.1	0,0009	0,0025	−2,12
229092_at	nuclear receptor subfamily 2, group F, member 2	NR2F2	chr15q26	0,0035	0,0042	−2,13
230561_s_at	chromosome 2 open reading frame 67	C2orf67	chr2q34	<0,0001	0,0016	−2,15
213424_at	KIAA0895 protein	KIAA0895	chr7p14.2	0,0011	0,0025	−2,20
223089_at	vezatin, adherens junctions transmembrane protein	VEZT	chr12q22	0,0010	0,0025	−2,22
214808_at	–	–	–	0,0003	0,0018	−2,23
228397_at	taurine upregulated gene 1	TUG1	chr22q12.2	0,0028	0,0042	−2,28
209120_at	nuclear receptor subfamily 2, group F, member 2	NR2F2	chr15q26	0,0033	0,0042	−2,36
225662_at	sterile alpha motif and leucine zipper containing kinase AZK	ZAK	chr2q24.2	0,0035	0,0042	−2,38
219381_at	chromosome 5 open reading frame 42	C5orf42	chr5p13.2	0,0003	0,0018	−2,38
204363_at	coagulation factor III (thromboplastin, tissue factor)	F3	chr1p22-p21	0,0048	0,0049	−2,38
220987_s_at	chromosome 11 open reading frame 17///NUAK family, SNF1-like kinase, 2	C11orf17///NUAK2	chr11p15.3///chr1q32.1	0,0035	0,0042	−2,43
209703_x_at	methyltransferase like 7A	METTL7A	chr12q13.13	0,0020	0,0036	−2,44
225946_at	Ras association (RalGDS/AF-6) domain family (N-terminal) member 8	RASSF8	chr12p12.3	0,0010	0,0025	−2,45
205020_s_at	ADP-ribosylation factor-like 4A	ARL4A	chr7p21-p15.3	0,0003	0,0018	−2,46
228106_at	chromosome 4 open reading frame 30	C4orf30	chr4p15.32	0,0030	0,0042	−2,46
214298_x_at	septin 6	SEP6	chrXq24	0,0050	0,0050	−2,49
215046_at	chromosome 2 open reading frame 67	C2orf67	chr2q34	0,0043	0,0046	−2,56
210869_s_at	melanoma cell adhesion molecule	MCAM	chr11q23.3	0,0034	0,0042	−2,63
218326_s_at	leucine-rich repeat-containing G protein-coupled receptor 4	LGR4	chr11p14-p13	0,0039	0,0044	−2,64
201505_at	laminin, beta 1	LAMB1	chr7q22	0,0002	0,0018	−2,65
209681_at	solute carrier family 19 (thiamine transporter), member 2	SLC19A2	chr1q23.3	0,0008	0,0025	−2,67
242691_at	–	–	–	0,0002	0,0018	−2,67
204004_at	PRKC, apoptosis, WT1, regulator	PAWR	chr12q21	0,0007	0,0025	−2,70
241879_at	–	–	–	0,0020	0,0036	−2,71
226374_at	–	–	–	0,0041	0,0044	−2,72
244043_at	–	–	–	0,0017	0,0034	−2,73
203627_at	insulin-like growth factor 1 receptor	IGF1R	chr15q26.3	<0,0001	0,0004	−2,78
227320_at	family with sequence similarity 101, member A	FAM101A	chr12q24.31	0,0030	0,0042	−2,80
200953_s_at	cyclin D2	CCND2	chr12p13	0,0040	0,0044	−2,80
227911_at	Rho GTPase activating protein 28	ARHGAP28	chr18p11.31	0,0033	0,0042	−2,88
1557293_at	hypothetical gene supported by AK128346	LOC440993	chr3q29	0,0010	0,0025	−2,92
203628_at	insulin-like growth factor 1 receptor	IGF1R	chr15q26.3	0,0003	0,0018	−3,02
212913_at	chromosome 6 open reading frame 26///mutS homolog 5 (E. coli)	C6orf26///MSH5	chr6p21.3///chr6p21.33	0,0030	0,0042	−3,81
226750_at	La ribonucleoprotein domain family, member 2	LARP2	chr4q28.2	0,0004	0,0022	−3,85
205848_at	growth arrest-specific 2	GAS2	chr11p14.3-p15.2	0,0017	0,0034	−4,19
202345_s_at	fatty acid binding protein 5 (psoriasis-associated)///fatty acid binding prote	FABP5///FABP5L2///FABP5L7	chr11q12.1///chr13q14.3///chr8q21.13	0,0011	0,0025	−6,37
230781_at	–	–	–	<0,0001	0,0018	−21,05

Genes have been selected by using a combined criterion which required a fold change (FC) of 2 or higher and a statistical significance of p<0.005 between subgroups.

### Functional Annotation Clustering of Differentially Expressed Genes

Over-represented common gene ontology Biological Function categories and clusters were identified in groups of differentially expressed genes using the Database for Annotation, Visualization and Integrated Discovery (DAVID v 6.7) [17], [18]. The analyses from each contrast were compared for recurring functional themes.

### Real-time RT-PCR Validation

The levels of expression of selected genes were quantified using real-time reverse transcription polymerase chain reaction (RT-PCR) analysis. Briefly, 300 ng total RNA was reverse transcribed with High Capacity RNA-to-cDNA Master Mix (Applied Biosystems). The genes were amplified using commercially available Taqman probes (Applied Biosystems, see Table S3) or custom Taqman probes designed using Primer Express software, version 2.0 (Applied Biosystems). The Taqman Gene Expression kit (Applied Biosystems) was used for real-time PCR analysis. The relative differences in expression between groups were expressed using cycle threshold (Ct) values and the ΔΔCt method [19] as follows. The Ct values of the genes were first normalized with glyceraldehyde 3-phosphate dehydrogenase of the same sample. Assuming that the Ct value is reflective of the initial starting copy and that there is 100% efficiency, a difference of one cycle is equivalent to a twofold difference in starting copy. Means and standard deviations (n = 4) for RT-PCR data were calculated. Statistical significance of the differences was evaluated by the Mann-Whitney test. The level of significance was set at p≤0.05.

## Results

### Tumor Characteristics and WHO Grades

The clinical metadata and histological findings for the fifty-four samples analyzed are reported in Table S1. All 54 meningioma samples studied here were classified into WHO meningioma grades I (40 cases) and grade II (14 cases) according to histopathological evaluation. Combined metabolic profiling, cytogenetics and FISH data for 34 of these meningiomas was previously reported [9] Patients were followed up for evaluating recurrence after surgery. Karyotyping was obtained in 37 cell cultures from 54 different cases. Sample material was adequate for FISH studies in 42 cases. All cases without chromosomal aberrations belonged to the histological grade I meningioma group (see Figure S1 in File S1). All cases with alterations in chromosome 22 as the only chromosomal anomaly were also of histological grade I. According to FISH chromosomal analysis, only 16% (5 out of 32) of benign meningiomas are −1p whereas this percentage rises to 70% (7 out of 10) in atypical meningioma (two-tailed Fisher test p-value of 0.023). All cases containing alterations in chromosome 14 were of histological grade II. Additionally, all grade II meningiomas showed a complex karyotype with 6 of them showing chromosomal alterations different to −1p, −22 and/or −14.

### Subgroups of Benign Meningioma

**Figure 1:**
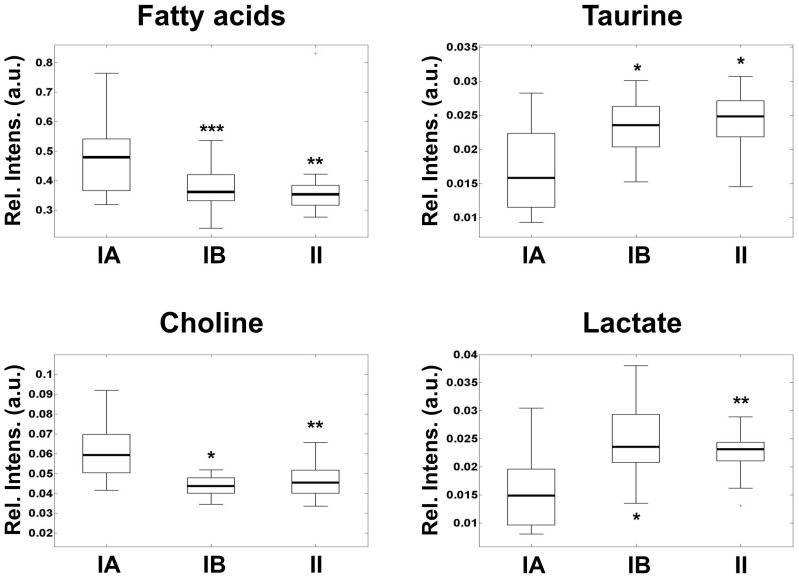
Box-and-whisker plots showing the levels of metabolic aggressiveness representative metabolites in benignA (box in the left), benignB (box in the middle) and atypical (box in the right) meningioma. Relative intensity levels are calculated as peak area normalized to total spectral area and expressed in arbitrary units. The boxes limits represent the lower and upper quartile limits. The median has been represented as a thicker line within the box. The whiskers are lines extending from each end of the boxes to show the extent of the rest of the data. Statistical significance with respect benignA levels are marked with stars (*, p<0.05; **, p<0.01; ***, p<0.005).

Cytogenetics and FISH analysis (Figure S1 in File S1) showed two clearly differentiated groups within the histopathological benign meningioma samples. These groups have been reported to have different metabolic behavior (benignA and benignB meninigioma [9]). According to these studies, classification of tumors in the benignB subgroup required the existence of chromosomal abnormalities detected either by FISH or by cytogenetics and/or levels of three out of four of the most representative metabolites (choline, lactate, taurine and fatty acids) within 2 standard deviations of the values for atypical meningioma reported. The benign tumors that did not fulfill these criteria were included in the benignA subgroup. The levels of choline, taurine, lactate and fatty acids were used to evaluate the degree of metabolic aggressiveness in the different subgroups (Figure 1). No other molecular correlation was detected with respect histological subtypes of benign meningioma, tumor location or tumor size (see Table S1). The metabolic profile of these samples allowed the detection of a subgroup of benign meningioma with metabolic aggressiveness and metabolism closer to atypical tumors. The metabolic profile of metabolic aggressiveness includes higher levels of phospholipids precursors, by-products of energy metabolism and cell antioxidants [9]. The meningioma subgroup benignA, containing 20 meningiomas, is defined by low metabolic aggressiveness and alterations in chromosome 22 as the only, if any, chromosomal anomaly. The meningioma subgroup benignB, which contained 20 meningiomas, includes tumors of histological grade I with additional chromosomal alterations. This meningioma subgroup exhibited higher metabolic aggressiveness than the subgroup benignA. In addition, metabolic levels in benignB meningioma were similar to those detected in atypical meningioma, suggesting increased clinical aggressiveness potential.

**Figure 2:**
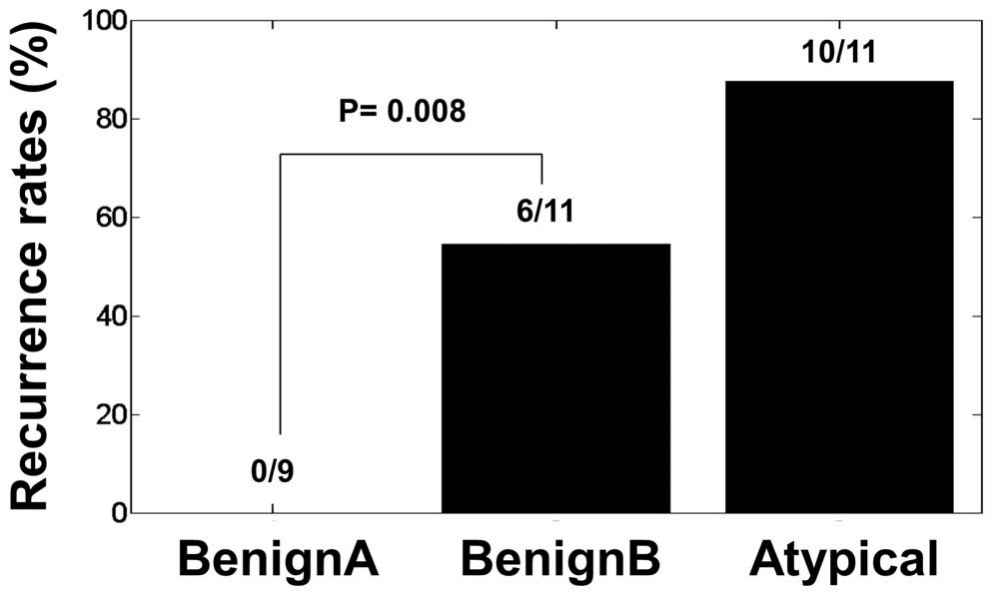
Bar graph showing tumor recurrence differences between benignA, benignB and atypical human meningioma at 4 years of follow up. Statistical significance in tumor recurrence between benignA and benignB meningioma as calculated by Pearson’s χ^2^ test is also shown.

Recurrence at 4 years was evaluated in a subset of 9 benignA, 11 benignB and 8 atypical meningioma patients for a preliminary assessment of potential clinical implications of these subgroups (see Figure 2 and Table S1). None of these patients with benignA meningioma exhibited recurrence after 4 years. Fifty percent (6 patients out of 11) of the patients with benignB meningioma show tumor recurrence in the 4 years after surgery. Ten out of eleven patients with atypical meningioma show tumor recurrence in the 4 years after surgery. The difference in tumor recurrence between benignA (none out of 9) and benignB (6 out of 11) meningioma was statistically significant (two-tailed Fisher test, p value 0.014). Gene expression differences between benignB meningiomas with and without recurrence were not significant.

### Genes Differentially Expressed in Meningioma Subgroups

**Figure 3:**
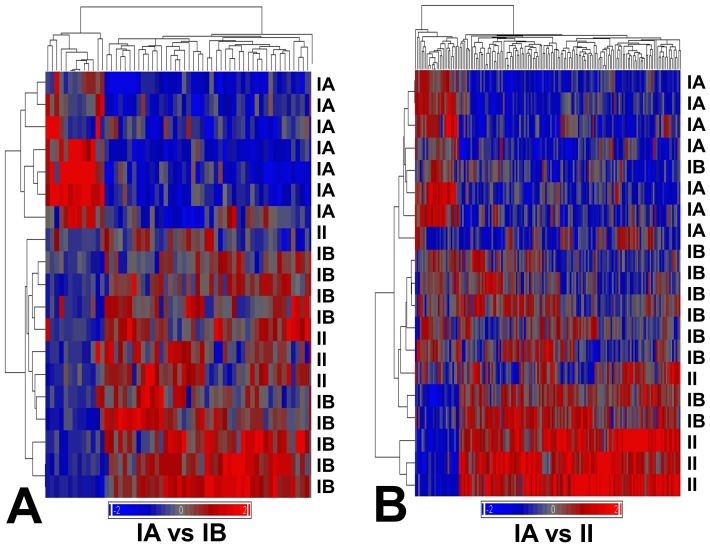
Unsupervised hierarchical cluster analysis of benign and atypical meningioma using genes differentially expressed between benignA and benignB meningioma subgroups (left, 59 genes) and benignA and atypical meningioma subgroups (right, 163 genes). Samples belonging to benignA (IA), benignB (IB and atypical (II) meningioma subgroups have been labeled accordingly. Genes have been selected by using a combined criterion, which required a fold change of 2 or higher and a statistical significance of p<0.005 between subgroups. The red and blue bars indicate genes demonstrating increased and decreased expression, respectively, based on the average relative fluorescence intensity values.

We examined expression profiles in benign and atypical meningioma for detecting gene markers of metabolic aggressiveness in benign meningioma. Only those tumors with sufficient fresh tissue remaining after diagnostic histopathology and metabolic profiling were selected for microarray expression profiling. Nineteen meningioma samples (7 benignA, 8 benignB and 4 atypical) underwent GeneChip analysis (data is available at http://www.ebi.ac.uk/arrayexpress/, accession code E-MEXP-3586). Four out of eight benignB meningioma analyzed showed recurrence after 4 years. We identified 59 genes differentially expressed between benignA and benignB meningioma subgroups (Table 1 and Figure 3A) and 163 genes differentially expressed between meningioma subgroups benignA and atypical (Table S2 and Figure 3B). The hierarchical clustering of the tumor samples according to these gene subsets showed that the gene signature of the benignB meningioma subgroup is between benignA and atypical meningioma. However, the distribution of samples, with more intermixing between benignB and atypical meningioma samples, suggests a profile closer to the atypical meningioma than to the benignA meningioma subgroup.

The number of genes differentially over-expressed in the different subgroups with respect the benignA meningioma was 46 in benignB meningioma (Table 1) and 136 in atypical meningioma (Table S2). Among them, IGF1R, MutS protein homolog 5 (MSH5), La-related protein 2 (LARP2), Growth arrest-specific protein 2 (GAS2), Fatty acid-binding protein, epidermal (FABP5) and probe 230781_at were over-expressed in benignB with respect benignA meningiomas with a fold change higher than 3. On the other hand, 13 genes in benignB meningioma (Table 1) and 27 genes in atypical meningioma (Table S2) were under-expressed with respect benignA meningioma. Homeobox protein Hox-B3 (HOXB3), LIM domain only protein 3 (LMO3), Homeobox protein Hox-B6 (HOXB6), Homeobox protein Hox-A3 (HOXA3) and DNA-binding protein inhibitor ID-2 (ID2) showed higher under-expression in benignB with respect benignA meningiomas with a fold change lower than −3. LMO3 was the gene showing the highest combined under-expression in both benignB and atypical meningioma with respect benignA meningioma. Probeset 230781_at exhibited the highest over-expression in both benignB and atypical meningioma with respect benignA meningioma. Some of the genes differentially expressed between subgroups were directly related to the metabolites selected for the evaluation of metabolic aggressiveness (Figure S2 in File S1).

Previous studies reported differential gene expression profiles in meningioma subtypes. However, the lists of genes differ significantly among the studies. A recent report used multiple data sets and restrictive criteria to identify genes differentially expressed between grade I benign and grade III anaplasic meningiomas [12]. Perez-Magan et al [13] also reported an association between meningioma recurrence and genes in transforming growth factor-β (TGF-β pathways. We evaluated the expression of the reported genes in our subgroups to explore the validity of these markers in the determination of metabolic aggressiveness in benign and atypical meningioma. Most of these genes exhibited statistically significant differences between the benignA and atypical meningioma subgroups (Table S2). None of them exhibited statistically significant differences between benignA and benignB meningioma.

### Analysis of Selected Genes by Real-time RT-PCR

**Figure 4:**
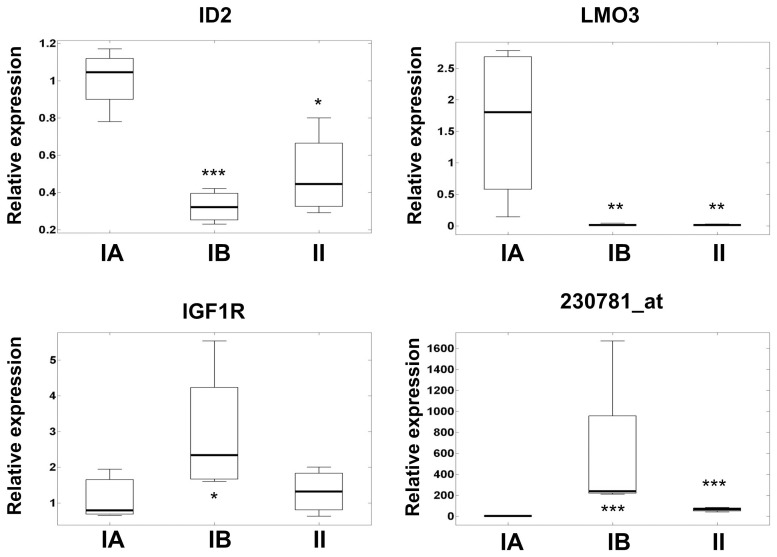
Box-and-whisker plots showing the relative expression levels measured by real-time RT-PCR for selected genes in benignA (4 samples, box in the left), benignB (4 samples box in the middle) and atypical (4 samples, box in the right) meningioma. The boxes limits represent the lower and upper quartile limits. The median has been represented as a thicker line within the box. The whiskers are lines extending from each end of the boxes to show the extent of the rest of the data. Statistical significance with respect benignA levels are marked with stars (*, p<0.05; **, p<0.01; ***, p<0.005).

Four genes were selected for further analysis by real-time RT-PCR in 12 of the analyzed samples for technical validation. We selected LMO3 and 230781_at because they show the highest under-expression and over-expression respectively in both benignB and atypical meningioma with respect benignA meningioma. We also selected IGF1R and ID2 because they were the most statistically significant genes in the sub-network most enriched (sub-network of expression targets of Transforming growth factor beta 1, TGFB1, Figure S3 in File S1) by the 59 genes differentially expressed between subgroups. Figure 4 shows the relative expression levels, measured by RT-PCR, for genes LMO3, ID2, IGF1R and the 230781_at probeset in benignA, benignB and atypical meningioma subgroups. The real-time RT-PCR data confirmed significantly higher mRNA levels of IGF1R in benignB with respect benignA meningioma. In line with the microarray data, real-time RT-PCR also showed statistically significant under-expression of LMO3 and ID2 in benignB and atypical meningioma with respect benignA meningioma. In fact, real-time RT-PCR results suggest much more decreased expression of LMO3 in benign and atypical meningioma than that extracted from the microarray multi-probe data.

## Discussion

Based on clinical and pathological findings, most meningiomas are considered slow-growing tumors surgically curable. Aggressiveness in meningioma is based solely on WHO grade and morphological parameters. However, up to 20% of the histologically benign meningiomas recur unexpectedly, even after complete resection, posing a challenge to the management of these tumors [2]. There is a need for identification of meningioma subgroups beyond conventional histological subtypes. A molecular definition of aggressiveness may provide additional criteria for the diagnosis of meningioma. Recent studies demonstrate more aggressive metabolism in meningiomas with chromosomal instabilities regardless of their histological grade [9]. Our results show that this subgroup of benign meningiomas with aggressive metabolism has a distinct gene expression profile and, more importantly, correlations with tumor recurrence. This gene expression profile partially resembles that of atypical meningioma. This subgroup of benign meningioma with both metabolic and gene expression profiles close to atypical meningioma exhibit higher rates of tumor recurrence than other benign meningioma.

Biochemical and metabolic changes of any cell population precede morphological and cellular changes. The detection of these molecular changes may help in the identification of new molecular subgroups for better management of the tumor patient. In this study, the use of molecular and genetic criteria for the definition of target subgroups provided differential expression levels with high statistical significance. These target subgroups exhibited statistically significant differences in tumor recurrence rates. Gene expression microarray technology allowed us to detect gene markers of metabolic and clinical aggressiveness in benign meningioma.

Cancer cells use an altered metabolism compared with that of normal differentiated adult cells in the body [20]. Tumors have high requirements for energy, substrates to grow and divide, and control of the redox potential and reactive oxigen species in the cell. The levels of all these metabolites establish the biosignature of what is called metabolic aggressiveness [9]. A tumor metabolically aggressive shows increased energy demand, higher hypoxic conditions, increased membrane turnover and cell proliferation and increased resistance to apoptosis. Although these changes are probably unspecific for many tumors, they represent a robust way to detect metabolically aggressive tumors. The gene expression profile of benign meningioma with aggressive metabolism reported here shows that some of the genes directly related to these metabolic processes are also altered. Choline-kinase beta gene expression (CHKB, Figure S2 in File S1) progressively increased in parallel to choline levels (Figure 1), suggesting increased choline phosphorylation in metabolically aggressive tumors. Phosphocholine and choline compounds are well established proliferation markers. Fatty acid binding protein 5 (FABP5) shows also increased expression for both benignB and atypical meningioma subgroups with respect benignA meningioma. This suggests increased mobilization of fatty acids, in line with the levels of fatty acids detected in the corresponding metabolic profiles. Similarly, Taurine up-regulated gene 1 (TUG1) exhibits higher expression levels in both benignB and atypical meningioma with respect benignA meningioma. Taurine is a metabolite essential in the response to stress and acts as a cellular antioxidant under certain conditions [21]. Previous studies suggest a potential anti-apoptotic role for taurine [22].

Growth factor receptors are over-expressed in many cancers [23]. Receptor over- expression may enable the cancer cell to become hyper-responsive to ambient levels of growth factors. Among the different growth factor, insulin-like growth factors (IGF) have important roles in cancer [24]. The IGF1R is a multi-functional tyrosine-kinase, which plays relevant roles in cell proliferation, differentiation, DNA repair, protection against apoptosis and, of course, metabolism. Previous studies suggest that the differential expression of IGF1R may be important in autocrine stimulation of brain tumor cell growth [25]. A recent study showed that inhibitors of IGF1R may have anti-tumoral effects in benign meningioma [26]. Our results by gene expression microarrays and real-time RT-PCR show that IGF1R is closely related to metabolic aggressiveness in histological benign meningioma but not in atypical meninhioma. IGF1R is over-expressed in the benign meningioma subgroup with higher rates of membrane turnover, higher energy demand and increased resistance to apoptosis (Figure 4). In atypical meningioma, on the contrary, levels of IGF1R are similar to those detected in the benign meningioma subgroup with lower metabolic aggressiveness. The interpretation of this fact is far from simple. The factors that influence IGF1R tumorigenic effects are not fully understood. For example, in the absence of insulin receptor substrate 1 (IRS1), IGF1R transmits a signal that promotes differentiation [27]. A microarray study on meningioma grade reported increased expression of IGF pathway genes, namely IGF2, IGFBP3 and AKT3, in meningiomas with losses on chromosomes 10 and 14 [28]. It seems that meningiomas progress by activating the IGF pathway among others. However, the way this is achieved in the benignB subgroup is different to that in atypical meningioma. Evidently, more research is needed to further clarify this result. Overall the differential expression observed for other genes downstream of insulin and insulin growth factors pathways suggests a central role in the development of metabolic aggressiveness in histological benign meningioma.

In this study, the gene showing the highest over-expression in correlation with metabolic aggressiveness belongs to a microarray probeset without annotation (230781_at). Sequence alignment of its target sequence reveal high homology with the hARD2 gene, as previously suggested [29]. hARD2 is a gene involved in several processes critical for tumor progression, like activation of hypoxia inducible factors [30]. Although RT-PCR analysis revealed that this probeset does not represent the hARD2 gene, the high sequence homology detected suggests a potential similar function for the hypothetical protein expressed by the 230781_at gene. Interestingly, this same probeset was reported as differentially expressed between genders in the course of normal brain aging [29]. Meningiomas are more common in females than in males. These facts suggest a potential additional role for the gene represented in probeset 230781_at in meningioma progression and deserves further investigation.

**Figure 5:**
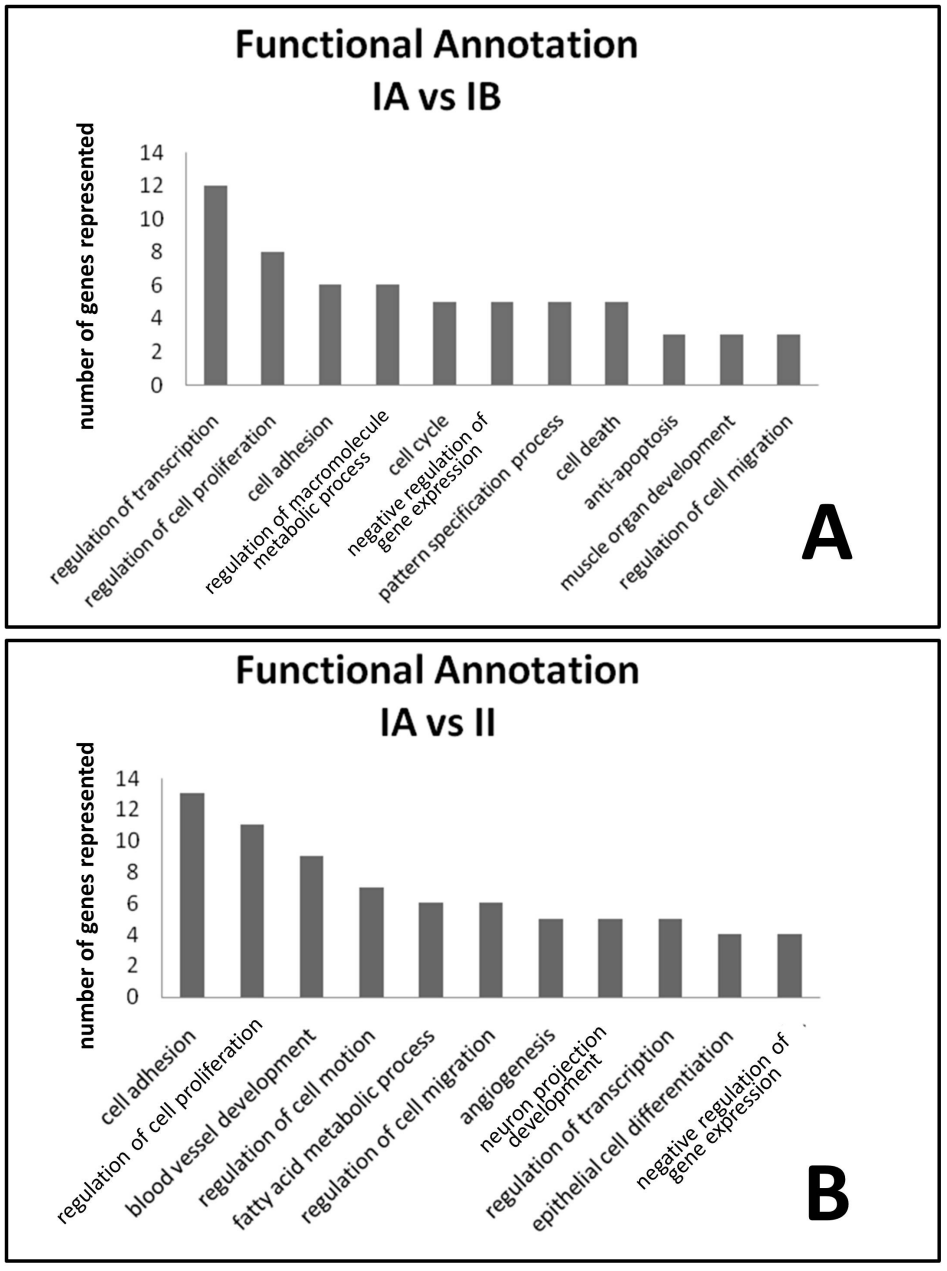
Distribution of GO classes differentially expressed between benignA and benignB meningioma subgroups (panel A, top) and benignA and atypical meningioma subgroups (panel B, bottom). GO classes populated by at least 4 genes differentially expressed are represented.

The use of curated databases, like Gene Ontology (GO) [31], for assigning functional categories to significant genes provides insight into gene classes and signaling pathways. We applied pathway analysis and GO functional annotation to genes differentially expressed between meningioma subgroups (Figure 4 and Figure S4 in File S1). Interestingly, the subnetwork most represented by the list of 59 genes reported here corresponds to the target gene of TGF-β. Previous studies have associated tumor recurrence and other genes closely related to this pathway [13]. TGF-β signaling also seems to have an inhibitory effect on meningioma proliferation [32]. Most populated GO categories in the comparison between subgroups are represented in Figure 4. Genes affecting regulation of transcription constitute the main difference between subgroups of benign meningioma (Figure 5A). Among all transcription factors, LMO3 showed the highest differences between benignA meningioma and more aggressive benignB and atypical meningioma (Figure 4). LMO3 belongs to the LIM-only group of transcriptional regulators, which have been implicated in cancer through its interactions with other transcription factors[33]–[35]. Although some studies report an oncogenic role for LMO3 in neuroblastoma, our results suggest that, in meningioma, this is only the case in the more benign forms. ID2 is another gene, closely related to transcription factors, which is highly down-regulated in metabolically aggressive meningioma (Figure 4). ID proteins are inhibitors of basic helix-loop-helix transcription factors and generally stimulate cell proliferation and inhibit differentiation [36]. However, previous works show that ID2 acts as an important protein for the maintenance of a differentiated and noninvasive phenotype in normal and transformed breast cells [37]. Regulation of transcription is represented to a much lesser degree in the differences between benignA and atypical meningioma than between benignB and atypical meningioma. The GO distribution for the differences between meningioma subgroups also include classes directly related to tumor vascularization and invasion, like angiogenesis, cell migration and cell adhesion. These classes are predominant in the differences between benignA and atypical meningioma but not in the differences between benignA and benignB meningioma. This GO distribution suggests that, first, regulation of transcription is an essential process in the development of metabolic aggressiveness in meningioma, and second, the activation of genes involved in vascularization and invasion is critical for meningioma progression towards higher histological grades.

### Conclusions

In summary, this study provides gene expression biosignatures of metabolic and clinical aggressiveness in histological benign meningioma. To our knowledge, this is the first time that distinct gene expression profiles are reported for benign meningioma molecular subgroups with clinical correlation. Our results show that metabolic aggressiveness in otherwise histological benign meningioma proceeds mostly through alterations in the expression of genes involved in the regulation of transcription, mainly the LMO3 gene. Genes involved in tumor metabolism, like IGF1R, are also differentially expressed in meningioma subgroups with higher rates of membrane turnover, higher energy demand and increased resistance to apoptosis. This work shows that there is a close link between metabolic aggressiveness, rates of tumor recurrence and regulation of transcription in meningioma progression.

## Supporting Information

File S1 **Content: Figure S1.** Distribution of chromosomal instabilities detected in benignA (white), benignB (gray) and atypical (black) meningioma meningioma according to combined cytogenetic and FISH analysis. **Figure S2**. Values absolutes of fluorescent intensity from Gene Chip probesets related to relevant metabolites for biochemical aggressiveness **Figure S3**. We found the most relevant pathways and enriched sub-networks using analyzed genes. All the pathway analysis was performed using the Pathway Studio v8.0 software (Ariadne Genomics) with the Resnet 8.0 database. The pathway diagram was further filtered to show only genes that were involved with cellular processes and diseases associated with our selected genes to aid interpretation of the pathway network. Figure S3 represents the subnetwork of genes most represented by the list of 59 genes differentially expressed between groups (9 genes, 17% of representation), which corresponds to the sub-network for the target expression of TGFB1. **Figure S4**. Pathway (genes interaction network) mapping of altered cellular processes and diseases using Pathway Studio v8.0 software. Genes and interactions are represented with associated cellular processes and diseases. **A.** Gene downregulated in benignB with respect benignA meningiomas with a fold change lower than −3 (red) are represented with associated cellular processes and diseases. B. Gene upregulated in benignB with respect benignA meningiomas with a fold change lower than 3 (blue). Symbol keys are the same as for figure S3.
(DOC)

Table S1 **Clinical data for the patients recruited and the tumor analyzed.**
(XLS)

Table S2 **Genes differentially expressed between meningioma IA and II.**
(XLS)

Table S3 **Taqman probes used for gene expression validation.**
(DOC)
