# Supplementary material for: Gene Expression Profiles of Metabolic Aggressiveness and Tumor Recurrence in Benign Meningioma
Source: PLoS One. 2013 Jun 28;8(6):e67291. doi: 10.1371/journal.pone.0067291 (PMC3696107; doi:10.1371/journal.pone.0067291)
Supplement: File S1 — Content: Figure S1. Distribution of chromosomal instabilities detected in benignA (white), benignB (gray) and atypical (black) meningioma meningioma according to combined cytogenetic and FISH analysis. Figure S2. Values absolutes of fluorescent intensity from Gene Chip probesets related to relevant metabolites for biochemical aggressiveness Figure S3. We found the most relevant pathways and enriched sub-networks using analyzed genes. All the pathway analysis was performed using the Pathway Studio v8.0 software (Ariadne Genomics) with the Resnet 8.0 database. The pathway diagram was further filtered to show only genes that were involved with cellular processes and diseases associated with our selected genes to aid interpretation of the pathway network. Figure S3 represents the subnetwork of genes most represented by the list of 59 genes differentially expressed between groups (9 genes, 17% of representation), which corresponds to the sub-network for the target expression of TGFB1. Figure S4. Pathway (genes interaction network) mapping of altered cellular processes and diseases using Pathway Studio v8.0 software. Genes and interactions are represented with associated cellular processes and diseases. A. Gene downregulated in benignB with respect benignA meningiomas with a fold change lower than −3 (red) are represented with associated cellular processes and diseases. B. Gene upregulated in benignB with respect benignA meningiomas with a fold change lower than 3 (blue). Symbol keys are the same as for figure S3. (DOC) [file pone.0067291.s001.doc]

**Figure S1**

**
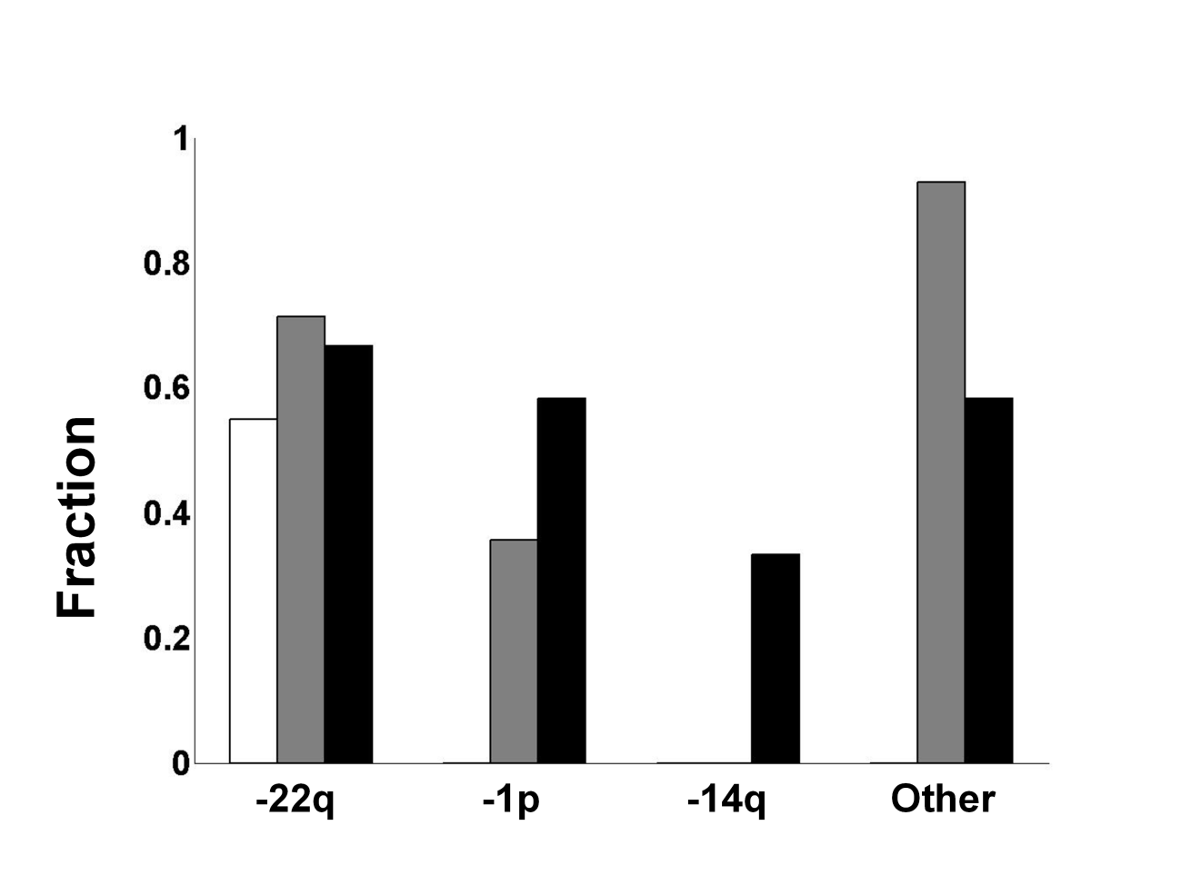
**

Figure S1. Distribution of chromosomal instabilities detected in benignA (white), benignB (gray) and atypical (black) meningioma meningioma according to combined cytogenetic and FISH analysis.

**Figure S2**


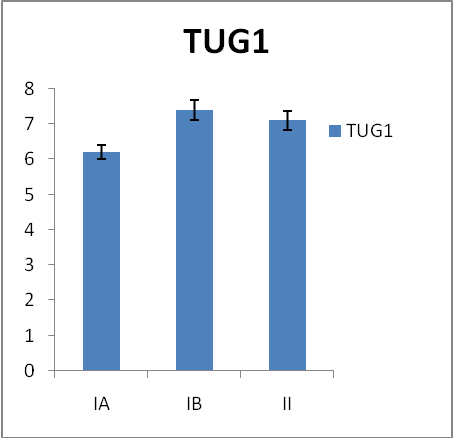


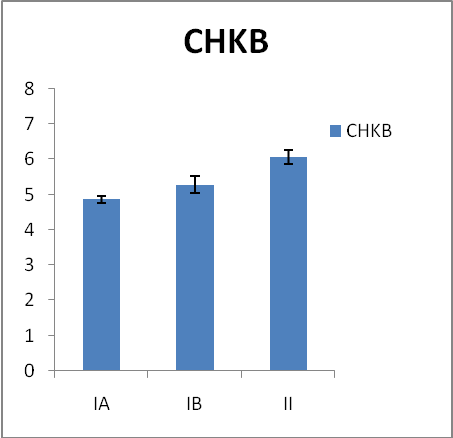


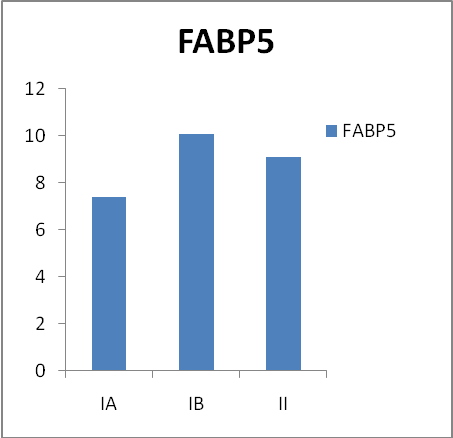


Figure S2. Values absolutes of fluorescent intensity from Gene Chip probesets related to relevant metabolites for biochemical aggressiveness.

**Figure S3**


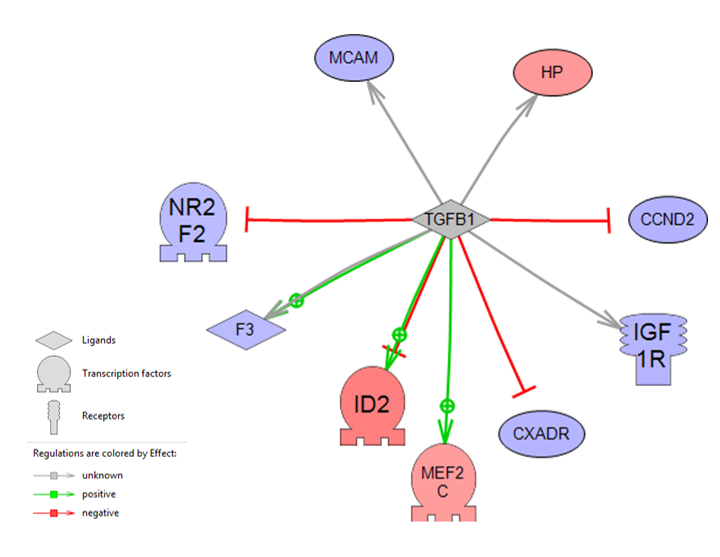


Figure S3. We found the most relevant pathways and enriched sub-networks using analyzed genes. All the pathway analysis was performed using the Pathway Studio v8.0 software (Ariadne Genomics) with the Resnet 8.0 database. The pathway diagram was further filtered to show only genes that were involved with cellular processes and diseases associated with our selected genes to aid interpretation of the pathway network. Figure S3 represents the subnetwork of genes most represented by the list of 59 genes differentially expressed between groups (9 genes, 17% of representation), which corresponds to the sub-network for the target expression of TGFB1.

**Figure S4**


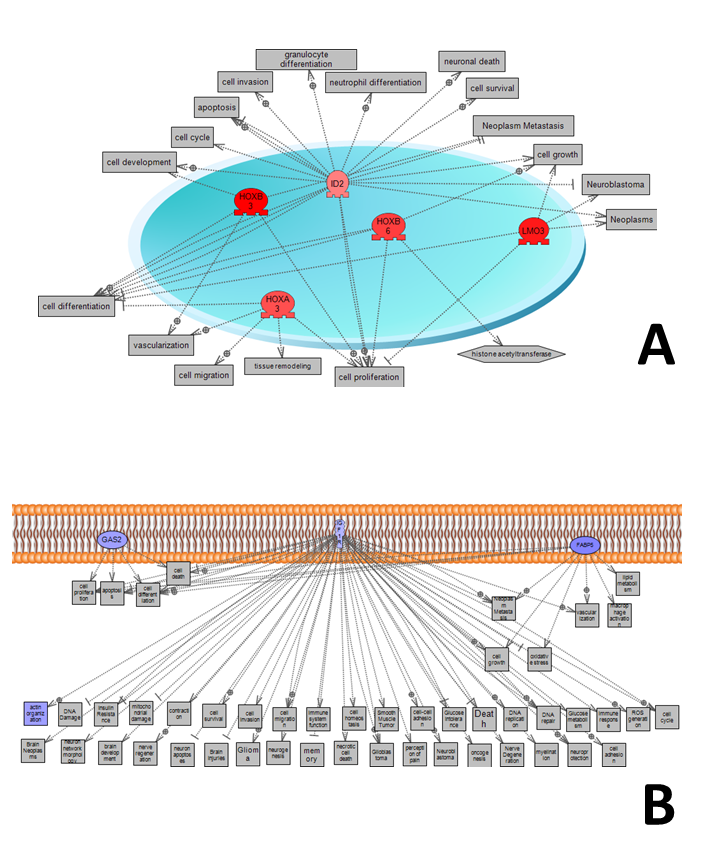


Figure S4. Pathway (genes interaction network) mapping of altered cellular processes and diseases using Pathway Studio v8.0 software. Genes and interactions are represented with associated cellular processes and diseases. **A.** Gene downregulated in benignB with respect benignA meningiomas with a fold change lower than -3 (blue) are represented with associated cellular processes and diseases. B. Gene upregulated in benignB with respect benignA meningiomas with a fold change lower than 3 (red). Symbol keys are the same as for figure S3.
