# Supplementary material for: Gene Expression Profiles of Metabolic Aggressiveness and Tumor Recurrence in Benign Meningioma
Source: PLoS One. 2013 Jun 28;8(6):e67291. doi: 10.1371/journal.pone.0067291 (PMC3696107; doi:10.1371/journal.pone.0067291)
Supplement: Table S3 — Taqman probes used for gene expression validation. (DOC) [file pone.0067291.s004.doc]

| **Table S3. Taqman Probes from Applied Biosystems Inc.** | | |
| --- | --- | --- |
| **Gene** | **TaqMan Probe ID** | **Amplicon lenght** |
| **LMO3** | Hs00375237_m1 | 82 |
| **Id2** | Hs00747379_m1 | 112 |
| **IGF1R1** | Hs00609566_m1 | 64 |
| **CUSTOM PROBE**  **230781_at** | AJWR1P7 ESERNA | ---- |
| **Control GAPDH** | PN4352934E | 122 |

RT-PCR amplification genes was performed using Taqman probes (Applied Biosystems) and normalized relative to a GAPDH reference gene. GAPDH gene expression was also measured using Taqman probe.
